# Maximal Cardiac Output Determines 6 Minutes Walking Distance in Pulmonary Hypertension

**DOI:** 10.1371/journal.pone.0092324

**Published:** 2014-03-19

**Authors:** Gaël Deboeck, Dolores Taboada, Guy Hagan, Carmen Treacy, Kathy Page, Karen Sheares, Robert Naeije, Joanna Pepke-Zaba

**Affiliations:** 1 Erasmus University Hospital, Department of Cardiology, Université Libre de Bruxelles, Brussels, Belgium; 2 Pulmonary Vascular Disease Unit, Papworth Hospital, Papworth, Cambridge, United Kingdom; 3 Department of Physiology, Faculty of Medicine, Université Libre de Bruxelles, Brussels, Belgium; University of Giessen Lung Center, Germany

## Abstract

**Purpose:**

The 6 minutes walk test (6MWT) is often shown to be the best predictor of mortality in pulmonary hypertension (PH) probably because it challenges the failing heart to deliver adequate cardiac output. We hypothesised that the 6MWT elicits maximal cardiac output as measured during a maximal cardiopulmonary exercise testing (CPET).

**Methods:**

18 patients with chronic thromboembolic pulmonary hypertension (n = 12) or pulmonary arterial hypertension (n = 6) and 10 healthy subjects performed a 6MWT and CPET with measurements of cardiac output (non invasive rebreathing device) before and directly after exercise. Heart rate was measured during 6MWT with a cardiofrequence meter.

**Results:**

Cardiac output and heart rate measured at the end of the 6MWT were linearly related to 6MW distance (mean±SD: 490±87 m). Patients with a high NT-pro-BNP achieve a maximum cardiac output during the 6MWT, while in normal subjects and in patients with a low-normal NT-proBNP, cardiac output at the end of a 6MWT was lower than achieved at maximum exercise during a CPET. In both cases, heart rate is the major determinant of exercise-induced increase in cardiac output. However, stroke volume increased during CPET in healthy subjects, not in PH patients.

**Conclusion:**

Maximal cardiac output is elicited by 6MWT in PH patients with failing right ventricle. Cardiac output increase is dependent on chronotropic response in patients with PH.

## Introduction

Pulmonary hypertension (PH) is a life-threatening disease characterized by an increase in pulmonary vascular resistance leading to symptoms and signs of right ventricular failure [1].

During exercise, cardiac output and oxygen consumption (VO_2_) increase linearly with work rate [2]. Therefore, the work rate or the speed of walking and VO_2_ are considered to reflect the ability of the heart to adapt flow output in response to exercise, and, in patients, the maximal distance walked in 6 minutes and maximal VO_2_ are thought to provide an indirect estimate of maximal cardiac output [3]. Among other field exercise tests, the 6MWT is shown to have the best ability to capture changes in exercise capacity [4] and has incidentally regularly been shown to be independent predictor of morbidity and mortality in PH [5]–[7]. We showed previously in PH patients that the 6-min walk test (6MWT) was performed with an oxygen consumption equivalent to VO_2_max arguing in favour of cardiac output limitation during 6MWT [8].

Therefore, the ability of the 6MWT to predict outcome should reside in the fact that it evaluates, like any exercise testing, the cardiac output reserve [3]. Unfortunately, measurement of cardiac output by invasive thermodilution technique is not feasible during walking and we found no data on cardiac output during 6MWT.

We therefore aimed to evaluate whether the 6MWT could be limited by cardiac output reserve in PH by using the inert gas rebreathing technique for determination of cardiac output (Innocor).

## Materials and Methods

### Ethical consideration

The study was accepted by the Cambridgeshire 1 Research Ethics Committee (ref: 08/H0306/104) and signed informed consent form was obtained prior to participation in the study.

### Patients

The study included 12 patients with chronic thromboembolic pulmonary hypertension (CTEPH), 2 with persistent pulmonary hypertension after pulmonary endarteriectomy and 6 with pulmonary arterial hypertension (PAH), all diagnosed following current guidelines [1] and 21 healthy controls. Three patients were in World Health Organisation functional class I, 8 in class II and 7 in class III. On the 18 patients, 5 were without treatment targeting the pulmonary circulation, 7 were treated with sildenafil, 2 with bosentan, 3 with sildenafil and bosentan, and 1 with intravenous epoprostenol. N-terminal fragment of pro- brain natriuretic peptide (NT-proBNP) level expressed in % of the higher negative predicted value (NPV) of heart failure [9] was 1209 (387–2267) (median (interquartile). Two patients presented with increased creatinine level (>1.2 mg/dL for men and >1.1 mg/dL for women) but presented with clinical and echocardiographic signs of right heart failure.

The eighteen patients (age: 50±14 y, height: 171±8 cm, weight: 74±18 Kg; sex 7/11 F/M) and 21 healthy subjects (age: 40±8 y, height: 170±10 cm, weight: 71±11 Kg sex 15/6 F/M) performed measurements of cardiac output before and directly after completion of a 6MWT on a 25 meters corridor (6MWT). All the patients and 10 healthy subjects (sex 5/5 F/M) performed a standard cardiopulmonary exercise testing (CPET) with measurement of cardiac output at rest, after 3 minutes of unloaded pedalling, and directly after completion of a maximal cardiopulmonary exercise testing (CPET).

NT-proBNP level of each patient was measured the day before exercise testing. All the patients were in sinus rhythm and none were on negatively chronotropic drugs.

### Assessment of cardiac output

Cardiac output was estimated by the alveolo-capillary transfer of nitrous oxide with use of sulfur hexafluoride as insoluble marker with an automated device (Innocor; Innovision, Odense, Denmark). The method has been previously shown to provide accurate measurements of pulmonary blood flow, and thus to provide an excellent approximation of cardiac output in the absence of pulmonary or cardiac shunts, in patients with heart failure or pulmonary hypertension, at rest and at exercise [10]–[13]. In the present study, patients with a shunt suspected on the basis of resting low arterial oxygenation estimated by pulse oximetry, or suddent increase in ventilation with decrease in end-tidal PCO_2_ [14] were excluded. Stroke volume (SV) was calculated by dividing cardiac output by heart rate. A nose clip was used to occlude the nostrils. Between each measurement a minimum washout period of 3 minutes was required before starting the next rebreathing manoeuvre. Cardiac output was measured after 5–10 min rest in dupli-triplicate.

### 6MWT

The 6MWT was performed according to standardised procedure [15]. Time was given every 2 minutes without encouragement. When the 6 minutes were elapsed the patient/subject was asked to come back at the same walking speed to the technician placed in the middle of the corridor and presenting the Innocor with a rubber tube mouthpiece. The patient/subject was then asked to stop walking, and, standing up, perform the rebreathing test immediately under the instructions of the experienced technician. Cardiac output measurements were obtained within 15–20 sec.

Pulse oximetry O_2_ saturation (SpO_2_) was measured permanently by a finger/ear probe (“AVANT 4000, Nonin medical, inc, Plymouth, MN USA). Twelve patients and 15 healthy subjects performed the 6MWT with a cardiofrequencemeter (Polar FT4, UK) and the maximal heart rate registered was taken into account to calculate stroke volume.

### CPET

Standard incremental cardiopulmonary exercise test until the symptom-limited maximum [16] was performed on an electronically braked bicycle. The CPET protocol consisted in an unloaded pedalling during the first 3 minutes and then a ramp increment of load of 5 to10 watts/min for the patients, 20–30 watts for healthy subjects. Ventilation and gas analysis was performed by breathing through a mouthpiece throughout the test (Oxycon pro, Viasys Healthcare, Germany). Heart rate and blood pressure were obtained via automatic standard ECG and sphygmomanometer. Cardiac output measurements were obtained at rest, after the 3 minutes of unloaded pedalling and immediately after the completion of maximal exercise capacity (cardiac output measurements were obtained within 15–20 sec of the end of the test). The Innocor device and the volume transducer were mounted in series allowing measurements to be performed easily with the same mouthpiece.

### Statistical analysis

Results are expressed as mean±SD except for Nt-proBNP level (% NPV of heart failure) expressed by median (interquartile). Comparison of cardiac output and stroke volume were performed using a paired t-test. Correlations were calculated by linear regression analysis. In graphics, line of identity represents same x-y coordinates.

## Results

Resting oxygen saturation was 98.5±1.5%. One patient was excluded from the study because he developed CPET criteria of shunting through a patent foramen ovale [14].

### 6MWT

The patients and the healthy subjects walked 490±87 m and 660±47 m respectively and achieved a heart rate of respectively 144±19 and 151±20 bpm.

### CPET

At the end of the CPET the maximal work load was respectively 80±29 and 214±69 watts for patients and healthy subjects, VO2peak was 17.2±4.3 and 35.2±9.2 mL/min.Kg, maximal heart rate (HRmax) was 149±24 and 172±13 bpm, maximal respiratory exchange ratio was 1.14±0.06 and 1.2±0.08 and VE/VCO2slope was 48±15 and 27±3.

### Cardiac output and stroke volume adaptation with 6MWT

**Figure 1:**
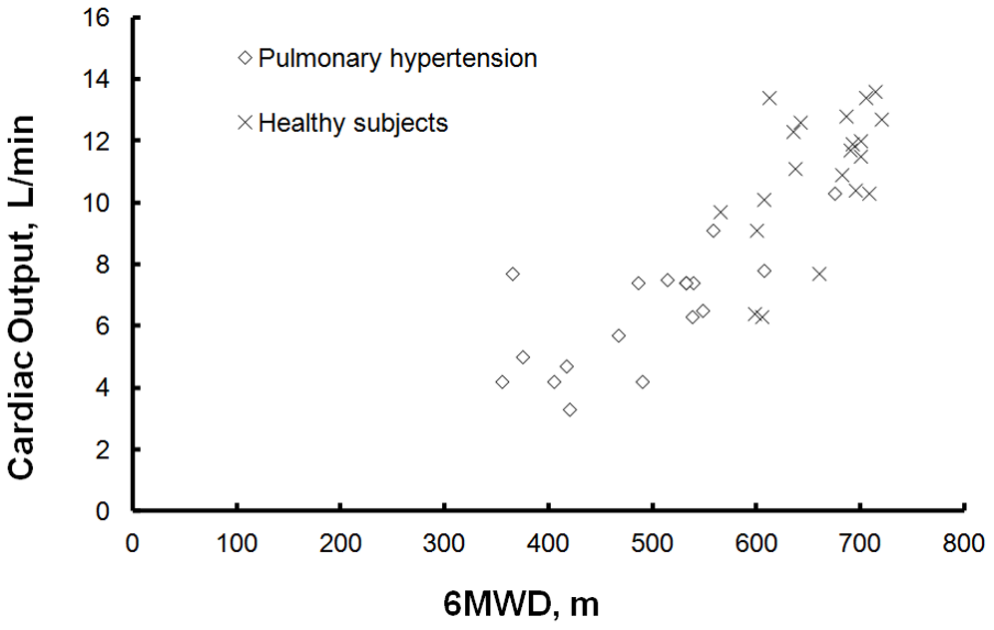
Cardiac output response to a 6MWT in relation with the 6MWD in 21 healthy subjects and 18 pulmonary hypertension patients.

With 6MWT, cardiac output (CO_6MWT_) increased from 3.8±0.7 to 6.5±1.9 L/min (P<0.001) in the patients and from 6.1±1.1 to 11±2.2 L/min (P<0.001) in the healthy subjects. The CO_6MWT_ was linearly related to 6MWD. (Fig 1)

Heart rate increased respectively from 76±11 to 144±19 bpm (P<0.001) and from 76±11 to 152±21 bpm (P<0.001) in patients and healthy subjects.

Heart rate was linearly related to 6MWD (r = 0.42, p = 0.029).

Stroke volume (SV_6MWT_) did not change in patients (from 52±8 to 45±10 mL (P = 0.054)) and decreased in healthy subjects (from 81±19 to 73±13 mL (P = 0.024)).

### Cardiac output and stroke volume during CPET

In one patient, the cardiac output was measured 1 minute before the end of the CPET and was considered to be maximal as the VO_2_ continued in plateau by then.

In patients, cardiac output (CO_CPET_) increased from 4.3±1.1 at rest to 5.4±1.6 at the end of the 3 minutes of unloaded pedalling (P<0.001) and to 7.2±2.2 L/min at peak exercise (vs unloaded pedalling, P <0.001). Maximal cardiac output correlated with VO_2_peak in ml/min (0.79, p <0.001) and in ml/min.Kg (0.58, p = 0.012).

In healthy subjects cardiac output increased from 6.6±1.3 at rest to 9.1±1.7 at the end of the 3 minutes of unloaded pedalling (P<0.001) and to 17.5±3 L/min at maximal exercise (vs unloaded pedalling, P<0.001).Maximal cardiac output correlated with VO_2_peak in ml/min (0.81, p = 0.005) and in ml/min.Kg (0.72, p = 0.019).

**Figure 2:**
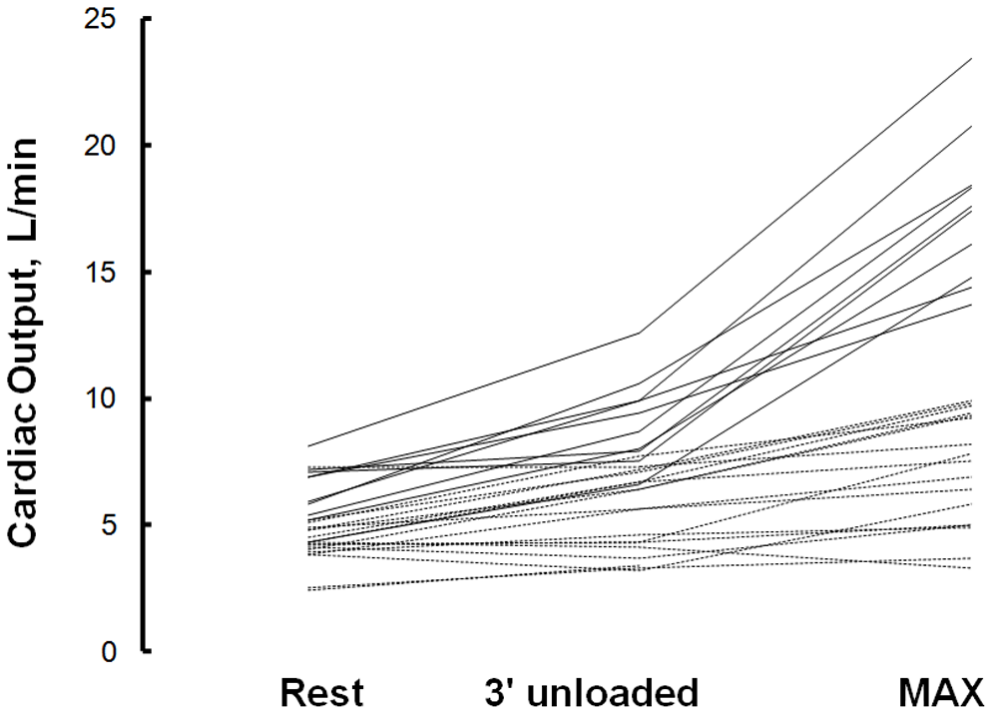
Cardiac output measured at rest, after 3 minutes of unloaded cycling and at directly after the end of maximal CPET in 18 pulmonary hypertension patients (stripped line) and 10 healthy subjects (solid line).

Individual changes are shown in figure 2.

In patients, stroke volume (SV_CPET_) did not change from 55±14 mL at rest to 56±16 at the end of the 3 minutes of unloaded pedalling (P = 0.781) and decreased to 48±14 mL at maximal exercise (vs 3 min unloaded pedalling, P<0.001). Peak heart rate was fairly correlated with VO_2_peak (r = 0.57, P<0.02).

**Figure 3:**
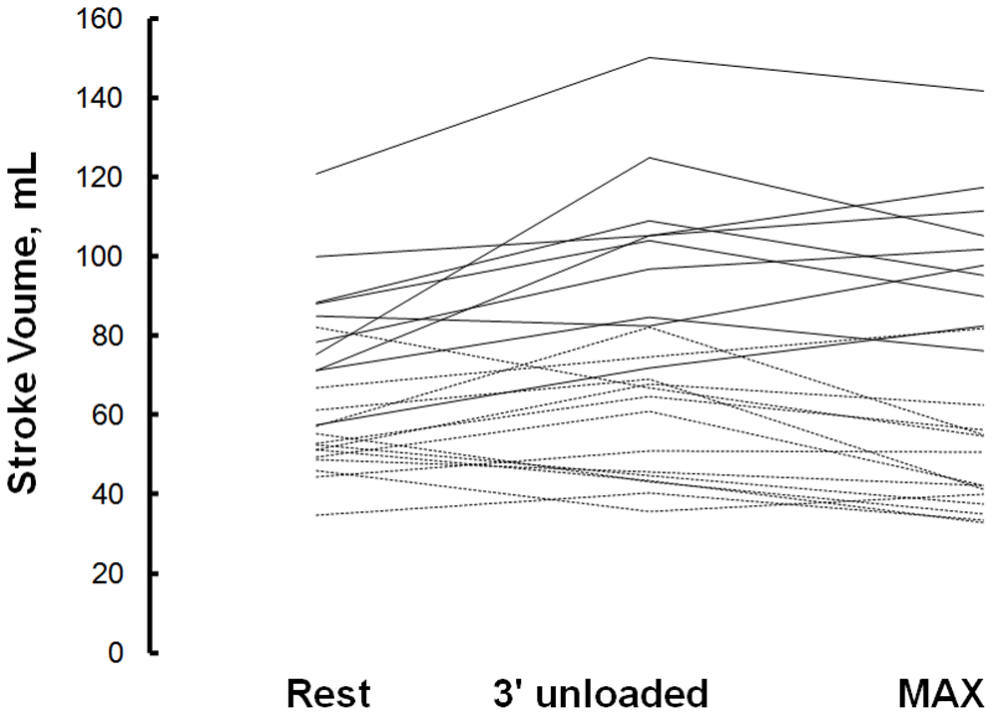
Stroke volume calculated at rest, after 3 minutes of unloaded cycling and directly after the end of maximal CPET in 18 pulmonary hypertension patients (stripped line) and 10 healthy subjects (solid line).

In healthy subjects, stroke volume increased from 84±17 mL at rest to 103±22 mL at the end of the 3 minutes unloaded pedalling (P = 0.002) and did not increase more at peak exercise (102±19 mL, vs 3 minute of unloaded pedalling, P = 0.724). Individual evolutions are shown in figure 3.

### Cardiac output and stroke volume: 6MWT vs CPET

**Figure 4:**
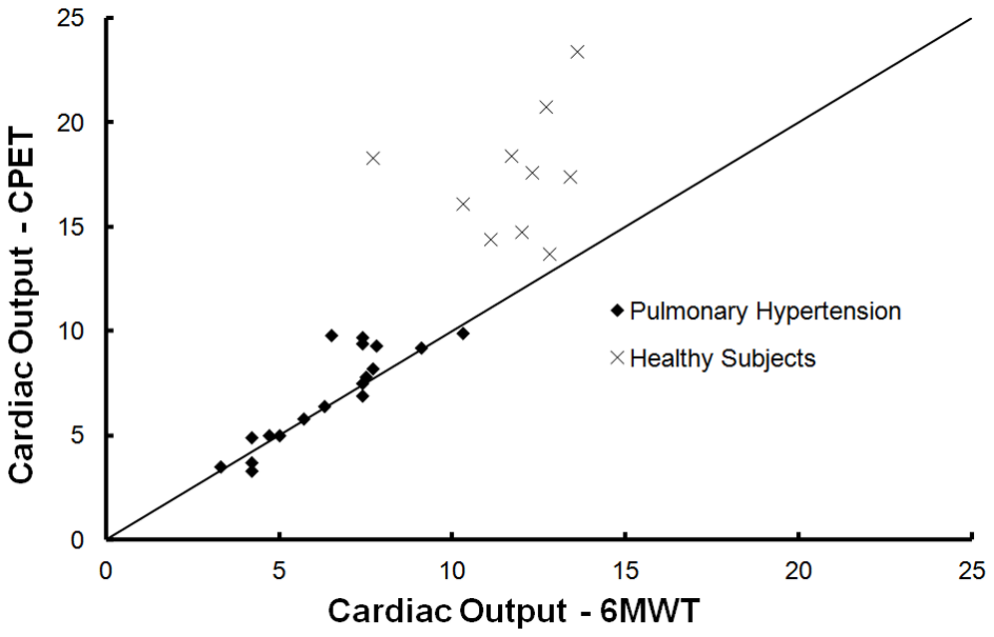
Cardiac output measured directly after the end of a 6MWT and of a CPET in 18 pulmonary hypertension patients and 10 healthy subjects. (-) Line of identity (where CO_6MWT_ = CO_CPET_).

**Figure 5:**
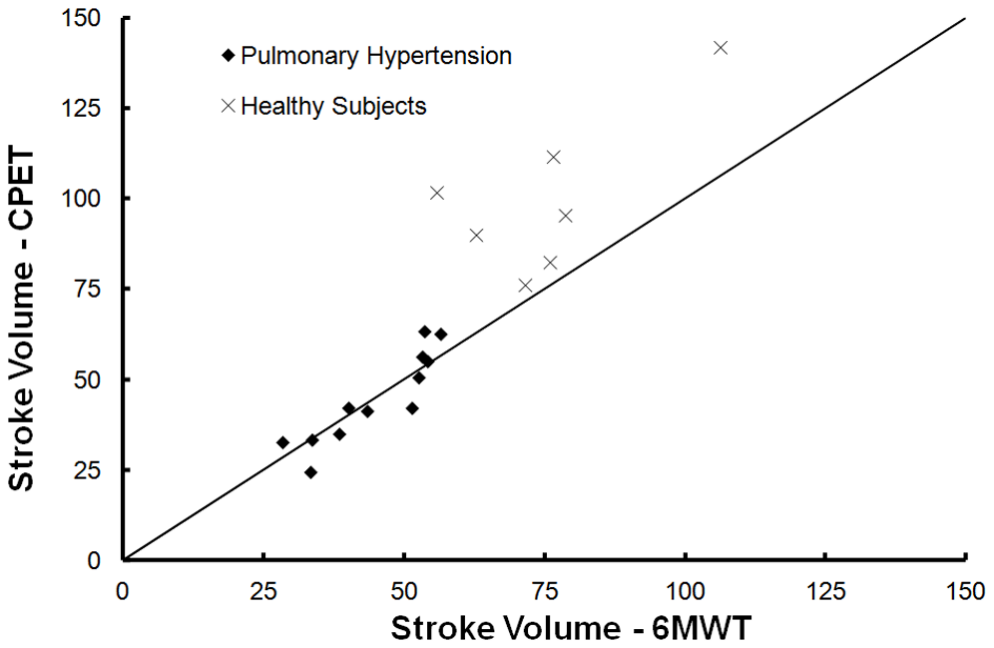
Stroke volume calculated directly after the end of a 6MWT and of a CPET in 12 pulmonary hypertension patients and 7 healthy subjects. (-) Line of identity (where SV_6MWT_ = SV_CPET_).

Figure 4 plots CO_CPET_ with CO_6MWT_ and figure 5 plots SV_CPET_ and SV_6MWT_ respectively.

**Figure 6:**
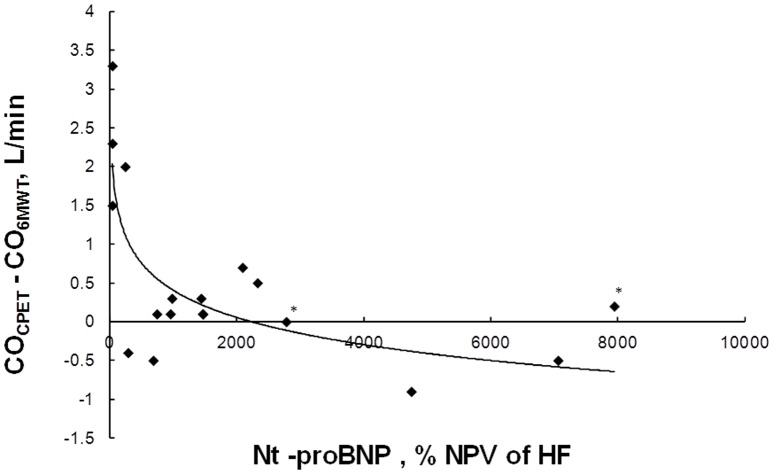
Difference between cardiac output measured at the end of a CPET and of a 6MWT in relation with Ntpro-BNP level in % of the highest negative predictive value for age in 18 PH patients (* indicate patients with high creatinine level).

Fourteen patients achieved a CO_6MWT_ within 1 L/min of the CO_CPET_ (6.2±2.1 vs 6.2±2.1 L/min (P = 0.952)) and the 4 remaining patients (2 with CTEPH and 2 with IPAH) achieved a higher CO_CPET_ (7.3±0.6 vs 9.6±0.2 L/min (P = 0.009)). NT-proBNP level (% NPV) was exponentially related with the difference between CO_CPET_ and CO_6MWT_ (CO_CPET_-CO_6MWT_) with the patients with higher difference having lowest % NPV for heart failure. (Figure 6)

Healthy subjects achieved a higher CO_CPET_ than CO_6MWT_ (P<0.001), however, one healthy subject had a higher CO_CPET_ by only 0.9 L/min.

Twelve patients performed the 6MWT with cardiofrequencemeter. They achieved similar SV_6MWT_ and SV_CPET_. (P = 0.997) Three of those patients had higher CO_CPET_, but SV_CPET_ differed from SV_6MWT_ by only 0.9, 3.1, and 6.1 mL.

Seven healthy subjects performed the 6MWT with a cardiofrequencemeter and presented with a higher SV_CPET_ (P<0.001). Two had a higher SV_CPET_ by only 4.6 and 6.5 mL.

## Discussion

The present results show that patients with a high NT-pro-BNP achieve a maximum cardiuac output during the 6MWT, while in normal subjects and in patients with a low-normal NT-pro-BNP, cardiac output at the end of a 6MWT is lower than achieved at maximum exercise during a CPET. In both cases, heart rate is the major determinant of exercise-induced increase in cardiac output. However, stroke volume increased during CPET in healthy subjects, not in PH patients.

The ability of the heart to increase cardiac output determines aerobic exercise capacity. In PH, cardiac output increase is dependent on the ability of the right ventricle to overcome high pulmonary vascular resistance [17]. With a failing right ventricle, maximum cardiac output decreases (Fig 2) and so does exercise capacity. Accordingly, maximum cardiac output was correlated with peakVO_2_ in both patients and healthy subjects. Maximal exercise testing provides therefore an indirect assessment of right heart failure. In that context the 6MWT is considered to be a good marker of exercise capacity which is in accordance with the linear relationship we found between 6MW distance and CO_6MWT_ (Fig 1) and relates most probably to the independent prognostic value of the 6MW distance [5]–[7]. However, the relationship between VO2p and 6MW distance is curvilinear with a steeper slope after 450–500 m indicating a lower sensitivity of the 6MWT to predict exercise capacity [18], [19]. In this present study the mean walking distance was high and the minimum distance walked was of 360 m (Fig. 1). In keeping with those previous studies, our results show a steep increase in CO_6MWT_ after approximately 450 m but it can be agreed that the relation will present with less steep slope below that threshold with therefore even more dependence of 6MWD to cardiac output.

We previously showed in PAH and chronic heart failure patients that maximal oxygen uptake was achieved during 6MWT arguing in favour of a cardiac output limitation [8], [20]. This is different pattern from healthy subjects achieving a VO_2_ during 6MWT of approximately 80% of VO_2_max [20]. In the present cohort of patients, CO_6MWT_ was similar to CO_CPET_ when NT-proBNP level was elevated (Fig. 6) probably meaning that a maximal cardiac output was achieved. Healthy subjects and patients with low-normal NT-proBNP achieved lower CO_6MWT_ than during CPET. Healthy subjects and patients without “failing” heart being able to move faster if allowed to run. It has moreover been shown that NT-pro BNP level correlates to 6MWD [21], and, in a post-hoc analyse of the TRIUMPH study, changes in 6MW distance were related to baseline NT-pro BNP level [22]. Combining NT-pro BNP level and 6MWD could be another clue analysing the predictive value of 6MWT.

In line with previous reports, our PH patients did not increase stroke volume with exercise [23], [24] supporting the fact that a high pulmonary vascular resistance affects right ventricle performance [23], [24]. Patients had indeed lower stroke volume than normal subjects with exercise, and none of our patients increased stroke volume with cardiopulmonary exercise test in contrast to our healthy subjects (Fig 5). Failing or not, the right ventricle of PH patients fails to maintain stroke volume, which is supported by the fairly good correlation between VO_2_peak and peak heart rate in our PH population (r = 0.56, P<0.02) and underlying the fact that exercise capacity in PH is highly dependent on a good chronotropic response [25], [26]. Chronotropic response has indeed been shown to be independent prognostic value in PH [27], [28].

In our healthy subjects, stroke volume expectedly increased with CPET. Stroke volume is usually thought to reach an asymptotic maximum when approaching maximal exercise [29]. However the pattern of the evolution of the SV during exercise has been differently reported [30], as we observed in our population. (Fig 3).

Stroke volume did not increase in patients or healthy subjects with 6MWT. (Fig 5) This is in line with previous findings for PH patients [26] but new information for healthy subjects. It was unexpected that healthy subjects would perform maximal walking distance without increasing their stroke volume. In accordance heart rate increased up to 97 and 88% of peak heart rate respectively and was linearly related to 6MWD. Moreover, we already showed that healthy subjects reach about 85% of peak heart rate during 6MWT [20]. Unfortunately HR was not obtained at 15–20 sec after 6MWT (during rebreathing measure). However a recalculation of stroke volume with an approximated decrease of HR of 10 beats did not change results.

A limitation of this study is that cardiac output was impossible to measure during 6MWT and was therefore measured directly after cessation of exercise. This could have altered genuine determination of exercise cardiac output as it is believed that cardiac output may rapidly decrease after cessation of exercise, especially when muscular venous return is not active anymore. However, in our settings cardiac output was determined directly after cessation of exercise (within 15–20 seconds) and in the same position for both exercise tests. We therefore believe that both conditions are comparable and that these measurements offer eventually good representation of physiological cardiac output response.

In conclusion, we showed that the 6MWT generates maximal cardiac output in PH patients with right heart failure and that may be the reason why it is so powerful predictor of mortality. We confirm that exercise cardiac output increase in PH is dependent on chronotropic response.

## References

[1] Hoeper MM, Bogaard HJ, Condliffe R, Frantz R, Khanna D, et al (2013) Definitions and diagnosis of pulmonary hypertension. J Am Coll Cardiol 62: D42–50. 24355641 10.1016/j.jacc.2013.10.032

[2] Stringer W, Hansen J, Wasserman K (1997) Cardiac Output estimated non-invsively from oxygen uptake (VO2) during exercise. J Appl Physiol 1997 82: 908–912. 10.1152/jappl.1997.82.3.908 9074981

[3] Naeije R (2010) The 6-min walk distance in pulmonary arterial hypertension: “Je t'aime, moi non plus”. Chest 137: 1258–60. 20525647 10.1378/chest.10-0351

[4] Mainguy V, Malenfant S, Neyron AS, Bonnet S, Maltais F, et al (2013) Repeatability and responsiveness of exercise tests in pulmonary arterial hypertension. Eur Resp J 42: 425–434. 10.1183/09031936.00107012 23100508

[5] Sitbon O, Humbert M, Nunes H, Parent F, Garcia G, et al (2002) Long-term intravenous epoprostenol infusion in primary pulmonary hypertension: prognostic factors and survival. J Am Coll Cardiol. 40: 780–788. 12204511 10.1016/s0735-1097(02)02012-0

[6] Groepenhoff H, Vonk-Noordegraaf A, Boonstra, Spreeuwenberg MD, Postmus PE, et al (2008) Exercise testing to estimate survival in pulmonary hypertension. Med Sci Sports Exerc. 40: 1725–32. 18799981 10.1249/MSS.0b013e31817c92c0

[7] Deboeck G, Scoditi C, Huez S, Vachiéry JL, Lamotte M, et al (2012) Exercise testing to predict outcome in idiopathic versus associated pulmonary arterial hypertension. Eur Respir J. 40: 1410–9. 22441747 10.1183/09031936.00217911

[8] Deboeck G, Niset G, Vachiery JL, Moraine JJ, Naeije R (2005) Physiological response to the six-minute walk test in pulmonary arterial hypertension. Eur Respir J 26: 667–672. 16204599 10.1183/09031936.05.00031505

[9] McMurray JJ, Adamopoulos S, Anker SD, Auricchio A, Böhm M, et al (2012) ESC Guidelines for the diagnosis and treatment of acute and chronic heart failure. Eur Heart J. 2012 Jul 33(14): 1787–847 doi: 10.1093/eurheartj/ehs104. Epub 2012 May 19 10.1093/eurheartj/ehs104 22611136

[10] Agostoni P, Cattadori G, Apostolo A, Contini M, Palermo P, et al (2005) Noninvasive measurement of cardiac output during exercise by inert gas rebreathing technique: a new tool for heart failure evaluation. J Am Coll Cardiol 46: 1779e81. 16256885 10.1016/j.jacc.2005.08.005

[11] Lang CC, Karlin P, Haythe J, Tsao L, Mancini DM (2007) Ease of noninvasive measurement of cardiac output coupled with peak VO2 determination at rest and during exercise in patients with heart failure. Am J Cardiol 99: 404e5. 17261407 10.1016/j.amjcard.2006.08.047

[12] Farina S, Teruzzi G, Cattadori G, Ferrari C, De Martini S, et al (2014) Noninvasive cardiac output measurement by inert gas rebreathing in suspected pulmonary hypertension. Am J Cardiol. Feb 1 113(3): 546–51. 10.1016/j.amjcard.2013.10.017 24315114

[13] Lee WT, Brown A, Peacock AJ, Johnson MK (2011) Use of non-invasive haemodynamic measurements to detect treatment response in precapillary pulmonary hypertension. Thorax. Sep 66(9): 810–4. 10.1136/thx.2011.159228 21700759

[14] Sun XG, Hansen JE, Oudiz RJ, Wasserman K (2002) Gas exchange detection of exercise-induced right-to-left shunt in patients with primary pulmonary hypertension. Circulation 105: 54–60. 11772876 10.1161/hc0102.101509

[15] American Thoracic Society (2002) ATS Statement: Guidelines for the six-minute walk test. Am J Respir Crit Care Med. 166: 111–117. 12091180 10.1164/ajrccm.166.1.at1102

[16] Palange P, Ward SA, Carlsen KH, Casaburi R, Gallagher CG, et al (2007) ERS Task Force, Recommendations on the use of exercise testing in clinical practice. Eur Respir J 29: 185–209. 17197484 10.1183/09031936.00046906

[17] Champion HC, Michelakis ED, Hassoun PM (2009) Comprehensive invasive and noninvasive approach to the right ventricle-pulmonary circulation unit: state of the art and clinical and research implications. Circulation 120: 992–1007. 19752350 10.1161/CIRCULATIONAHA.106.674028

[18] Lipkin DP, Scriven AJ, Crake T, Poole-Wilson PA (1986) Six minute walking test for assessing exercise capacity in chronic heart failure. Br Med J (Clin Res Ed) 8 292: 653–5. 10.1136/bmj.292.6521.653 PMC1339640 3081210

[19] Frost AE, Langleben D, Oudiz R, Hill N, Horn E, et al (2005) The 6-min walk test (6MW) as an efficacy endpoint in pulmonary arterial hypertension clinical trials: demonstration of a ceiling effect. Vascul Pharmacol 43: 36–9. 15890561 10.1016/j.vph.2005.03.003

[20] Deboeck G, Van Muylem A, Vachiéry JL, Naeije R (2013) Physiological response to the 6 minute walk test in chronic heart failure patients versus normal subjects. Eur J Prev Cardiol Mar 19 [Epub ahead of print] 10.1177/2047487313482283 23513011

[21] Fijalkowska A, Kurzyna M, Torbicki A, Szewczyk G, Florczyk M, et al (2006) Serum N-terminal brain natriuretic peptide as a prognostic parameter in patients with pulmonary hypertension. Chest 129: 1313–1321. 16685024 10.1378/chest.129.5.1313

[22] Frantz RP, McDevitt S, Walker S (2012) Baseline NT-proBNP correlates with change in 6-minute walk distance in patients with pulmonary arterial hypertension in the pivotal inhaled treprostinil study TRIUMPH-1. J Heart Lung Transplant 31: 811–6. 22759797 10.1016/j.healun.2012.04.005

[23] Nootens M, Wolfkiel CJ, Chomka EV, Rich S (1995) Understanding right and left ventricular systolic function and interactions at rest and with exercise in primary pulmonary hypertension. Am J Cardiol 75: 374–377. 7856531 10.1016/s0002-9149(99)80557-8

[24] Laskey WK, Ferrari VA, Palevsky HI, Kussmaul WG (1993) Pulmonary artery hemodynamics in primary pulmonary hypertension. J Am Coll Cardiol 21: 406–412. 8426005 10.1016/0735-1097(93)90682-q

[25] Deboeck G, Niset G, Lamotte M, Vachiéry JL, Naeije R (2004) Cardiopulmonary exercise testing in pulmonary arterial hypertension and in congestive heart failure. Eur Respir J 23: 747–751. 15176691 10.1183/09031936.04.00111904

[26] Provencher S, Chemla D, Herve P, Sitbon O, Humbert M, et al (2006) Heart rate responses during the 6 min walk test in pulmonary arterial hypertension. Eur Respir J 27: 114–120. 16387943 10.1183/09031936.06.00042705

[27] Wensel R, Opitz CF, Anker SD, Winkler J, Höffken G, et al (2002) Assessment of survival in patients with primary pulmonary hypertension. Importance of cardiopulmonary exercise testing. Circulation 106: 319–324. 12119247 10.1161/01.cir.0000022687.18568.2a

[28] Wensel R, Francis DP, Meyer FJ, Opitz CF, Bruch L, et al (2013) Incremental prognostic value of cardiopulmonary exercise testing and resting haemodynamics in pulmonary arterial hypertension. Int J Cardiol 167: 1193–8. 22494868 10.1016/j.ijcard.2012.03.135

[29] Guyton AC, Hall JE (2011) Textbook of Medical Physiology (12th ed.). Philadelphia, Pa.: Saunders/Elsevier.

[30] Vella CA, Robergs RA (2005) A review of the stroke volume repsonse to upright exercise in healthy subjects. Br J Sports Med 39: 190–195. 15793084 10.1136/bjsm.2004.013037 PMC1725174

